# Ocular Complications of Radiotherapy in Uveal Melanoma

**DOI:** 10.3390/cancers15020333

**Published:** 2023-01-04

**Authors:** Mihail Zemba, Otilia-Maria Dumitrescu, Alina Gabriela Gheorghe, Madalina Radu, Mihai Alexandru Ionescu, Andrei Vatafu, Valentin Dinu

**Affiliations:** 1 Department of Ophthalmology, University of Medicine and Pharmacy “Carol Davila”, 050474 Bucharest, Romania; 2 Department of Ophthalmology, ‘Dr. Carol Davila’ Central Military Emergency University Hospital, 010825 Bucharest, Romania; 3 Bucharest Emergency Eye Hospital, 030167 Bucharest, Romania

**Keywords:** uveal melanoma, radiation therapy, brachytherapy, proton beam therapy, stereotactic radiosurgery, stereotactic radiotherapy, complications

## Abstract

**Simple Summary:**

Radiation therapy, comprising brachytherapy, proton beam therapy, stereotactic radiotherapy, and radiosurgery, is the most used eye-sparing treatment for uveal melanoma worldwide. However, radiotherapy is associated with many complications, some of which are vision-threatening. Being aware of the different adverse effects of radiotherapy, the risk factors for their occurrence, their prognosis, and the available treatments for each complication is essential in aiding both the practitioner and patient to make the choice regarding the most appropriate therapy.

**Abstract:**

Uveal melanoma is the most common primary malignant intraocular tumor in adults. Radiation therapy has replaced enucleation and is now the preferred treatment in most cases. Nonetheless, around 70% of patients develop radiation-related complications, some of which are vision-threatening. The objective of this review is to present the most important complications associated with radiotherapy in the treatment of uveal melanoma and their pathogenesis, incidence, risk factors, and available preventive and therapeutic measures. The most common complications are cataracts, with a reported incidence ranging from 4% to 69%, and radiation retinopathy, reported in 5–68% of cases. Radiation-related complications are responsible for approximately half of secondary enucleations, the leading cause being neovascular glaucoma. A poor visual outcome is mainly associated with the presence of radiation retinopathy and radiation optic neuropathy. Therapeutic options are available for the majority of complications with the notable exception of optic neuropathy. However, many studies report a final visual acuity of less than 20/200 in more than 60% of treated eyes. Reducing complication rates can be achieved by lowering the dose of radiation, with the use of eccentric, customized plaques and careful planning of the irradiation delivery in order to protect structures vital to vision and by associating radiation therapy with other methods with the aim of reducing tumor volume.

## 1. Introduction

Uveal melanoma is the most common primary intraocular malignant tumor in adults [1]. The results of the Collaborative Ocular Melanoma Study (COMS) proved the nonsuperiority of enucleation compared to I-125 brachytherapy in terms of metastasis rates and survival and paved the way to a new era in the treatment of uveal melanoma for eye-sparing therapies. The most utilized globe-preserving treatment is radiation therapy, comprising ruthenium and iodine brachytherapy, proton beam therapy, stereotactic radiosurgery, and stereotactic radiotherapy [2]. All types of radiotherapy, if used as indicated, achieve good local tumor control and eye preservation rates [3,4,5]. However, the incidence of radiation-related complications is elevated, estimated to be between 61 and 78% [6,7]. Some complications lead to significant ocular morbidity, visual loss, and, in some cases, even secondary enucleation. The majority of complications are related to tumor characteristics and irradiation parameters [8]. This review aims to present the most important complications of radiation therapy encountered in uveal melanoma and their pathophysiology, incidence, risk factors, and available treatments and preventive measures, information that is essential in selecting the most appropriate therapy for each patient.

## 2. Materials and Methods

We conducted a comprehensive literature search in the MEDLINE electronic database using the PubMed interface. The word combinations used in the searching process were “uveal melanoma” AND “complications” AND, in turn, all of the following: “radiotherapy”, “radiation therapy”, “brachytherapy”, “proton beam therapy”, “radiosurgery”. Inclusion criteria consisted of articles written in English and regarding human pathology that were published prior to September 2022. We evaluated the title and abstract and retained the studies that investigated complications associated with the radiation therapies available at present for the treatment of uveal melanoma as well as studies regarding epidemiologic, pathophysiologic, and therapeutic aspects related to uveal melanoma and irradiation in this setting. Additional references were selected from the reference lists of the already retained studies. We excluded duplicates, studies not relevant to the topic, studies on animal models as well as conference presentations, editorials, letters to editors, and comments. After applying the inclusion and exclusion criteria, we retained 78 articles dating from 1996 to 2021.

## 3. Results

### 3.1. Uveal Melanoma

Uveal melanoma (UM) represents about 5% of all melanomas [9]. It arises from the melanocytes found in the uveal tract and has a particular molecular pathogenesis that distinguishes it from skin melanoma [1]. UM can originate in any of the components of the uveal tract, namely, the iris, the ciliary body, and the choroid. The incidence of UM is 5.1 cases per million worldwide, and it has remained stable over the years [1]. UM is more frequent in Caucasians and with older age, with a peak at 70 years [9].

Iris melanomas comprise approximately 5% of all uveal melanomas and are more common in younger patients [10]. They rarely metastasize and, thus, usually carry the best prognosis of the three. Ciliary body melanomas, on the other hand, have the worst prognosis, as they are generally more aggressive [11]. Moreover, they are diagnosed relatively late because of their location, which renders them asymptomatic and hidden from examination until late in their evolution [1,11]. The majority of UM, around 85%, arise in the choroid [1]. Anterior tumors have a worse prognosis because they can remain asymptomatic for long periods and because they can invade the ciliary body [11].

The COMS [12] classified choroidal melanomas according to their dimensions into:

- Small: 1.5–2.4 mm in height and 5–16 mm in diameter;
- Medium: 2.5–10 mm in height and ≤16 mm in diameter;
- Large: >10 mm in height and >16 mm in diameter.

An extraocular extension is rare, reported at around 15%, and may occur via the sclera, the optic nerve, or the vortex veins [13]. Metastases occur in almost half of the cases with by far the most important site for metastases being the liver (93%) followed by the lungs (24%) and the skeletal system (16%) [14].

The purpose of treatment is tumor clearance and prevention of metastases while preserving vision [15]. The choice of treatment is based on several factors, such as tumor location and size, the presence of metastases, patient comorbidities, and preference [16]. Treatment options include transpupillary thermotherapy; radiation therapy, comprising ruthenium 106 (Ru-106) and iodine 125 (I-125) brachytherapy; proton beam therapy (PBT), stereotactic radiosurgery (SRS), and stereotactic radiotherapy (SRT); and surgery, comprising endo- and exoresection and enucleation [2]. Although enucleation was historically the standard of care for uveal melanoma, ever since the results of the COMS Group showed no difference in the metastasis and survival rates between patients treated with I-125 brachytherapy and enucleation, the trend shifted towards eye-sparing therapies [12,17]. Local control of the tumor is reasonable. However, once metastases occur, the prognostic becomes poor, as the response to therapy of metastatic disease is usually mediocre [18].

### 3.2. Radiation Therapy in Uveal Melanoma

#### 3.2.1. Mechanism of Action

Radiation therapy is tumoricidal through several mechanisms. Firstly, ionizing radiation sterilizes and kills tumor cells either by directly damaging cells (through disruption of cellular membranes and organelles or alteration of the DNA) or indirectly via the production of free radicals, which are cytotoxic. Secondly, radiotherapy leads to the loss of endothelial cells and consecutive capillary occlusion which in turn results in ischemia of the neoplastic tissue. Thirdly, it helps expose tumor antigens to the immune system and thus enhances the specific antitumoral response [19]. Cell death may ensue as a result of the loss of the ability to divide followed by senescence and finally death through abnormal and fatal mitosis, apoptosis, or necrosis. Unlike the other two mechanisms, after necrosis, intracellular substances from tumor cells are released into the surrounding tissues, causing damage and inflammation [19]. Inflammation ensues early on after irradiation and is later followed by fibrosis [3].

#### 3.2.2. Types of Radiation Therapy in Uveal Melanoma—Brachytherapy

Brachytherapy is a type of internal therapy in which a plaque with a radioactive source is sutured to the global surface (episclera) and kept in place for 2–7 days depending on the isotope used [3]. The usual dose applied to the tumor apex is between 62 and 100 Gy [3,18,20]. The most used isotopes today in the treatment of UM are Ru-106 in Europe and I-125 in North America [8]. Ru-106 brachytherapy emits beta radiation and is indicated for tumors up to 5 mm in height [20]. I-125 is a gamma-emitting radioisotope with greater penetration and can thus be used in tumors thicker than 5 mm [18]. Brachytherapy is usually indicated for the treatment of small- and medium-sized tumors, but there are authors who report its use in the case of large tumors [21]. The plaque is customized to the tumor dimension, shape, and location with theoretical 2 mm safety margins. Contraindications for brachytherapy include large tumors and tumors within 2 mm from the optic disc [22]. Brachytherapy is more easily available than PBT, but the main disadvantage of plaque therapy is the reduced adaptability of the applicator to the specific area of interest, which can lead to unnecessarily irradiated tissue [15]. Overall efficiency in local tumor control is reported at rates of 84% [20]. Determinants of response to therapy are the radiation dose applied to the tumor apex and tumor location [20]. If the tumor is located at the posterior pole, close to the fovea or the optic nerve, there is a risk of damage to these structures when using brachytherapy, and the most suitable treatment is PBT [15].

#### 3.2.3. Types of Radiation Therapy in Uveal Melanoma—Proton Beam Therapy

Proton beam therapy is a form of external radiotherapy. PBT is indicated in the treatment of larger tumors with a height of more than 5 mm, tumors with a narrow base, tumors close to the optic nerve, and when there is ciliary body involvement of more than one clock hour or extrascleral extension [11,15]. It is also the best choice in the treatment of iris and ciliary body melanomas if surgery is not indicated, as it delivers less radiation to the cornea, sclera, and lens [15]. In PBT, a dose of around 56 Gy is delivered to the tumor over 4 days [18]. PBT requires the insertion of tantalum markers to mark the tumor and safety margins. Tantalum marker placement is easier than plaque placement and computer adjustments are possible afterward. Moreover, the beam can be adjusted to fit the tumor shape and to adapt the safety margins so that structures vital to vision are protected [15]. Results with PBT include local control rates in more than 90% of cases 5 years after treatment and survival rates without metastases between 72 and 90% [5,11]. However, PBT is only available in a few centers worldwide [3].

#### 3.2.4. Types of Radiation Therapy in Uveal Melanoma—Stereotactic Radiotherapy and Radiosurgery

Stereotactic radiotherapy and stereotactic radiosurgery involve the precise application of radiation to the tumor with an abrupt decrease in dosage at the margins so that surrounding tissues are protected. In the case of SRS, very high levels of radiation are applied simultaneously to a small area to ensure precise irradiation in a single-session treatment. SRT allows the delivery of lower levels of radiation at different times and to larger areas, thus being useful in treating very large tumor volumes. Multiple beams concomitantly deliver radiation to tissues from many different angles [23]. Careful customized preoperative planning is crucial for achieving the highest irradiation dose to the tumor and the lowest to critical structures, such as the optic nerve and the macular region. There are multiple available systems, including Gamma Knife (Elekta AB, Stockholm, Sweden), Cyberknife (Accuray Inc., Sunnyvale, CA, USA), and other linear accelerator (LINAC) systems (e.g., BrainLab, Novalis, TX, USA). Reported tumor control rates after SRS are 84–100% with eye preservation in 78–97.4% of cases [24].

#### 3.2.5. Recurrences after Radiation Therapy

Recurrences may have multiple mechanisms. The most frequent is an inadequate dose of radiation at the margins of the lesion, which results in recurrence at that precise location. Tumor cells may spread via an exudative retinal detachment and give rise to neoplastic tissue in the inferior peripheral retina. Late recurrences may also be due to radio resistance [25]. Tumor locations near the optic disc or the fovea are associated with a higher risk of local recurrence after Ru-106 brachytherapy [20]. Radiation therapy can be used together with surgery or transpupillary thermotherapy in order to increase local tumor control rates and limit the occurrence of complications, for example, in cases where the histology shows high-risk characteristics [15].

### 3.3. Complications of Radiation Therapy

The main predictors for the occurrence of complications after radiation therapy are tumor thickness and location and radiation dose [8]. With the exception of cataracts, most complications appear to be more frequent in younger patients [26]. The reported incidence and risk factors for some of the most important complications are summarized in Table 1 and Table 2, respectively.

#### 3.3.1. Ocular Surface

Ocular surface complications comprise radiation-related dry eye, conjunctivitis, and keratitis, which are usually superficial and punctate in nature [29]. These complications are more common after PBT than after brachytherapy. After iris melanoma was treated with stereotactic radiosurgery, keratitis was reported in 62.5% of cases [23]. Histological changes noted after plaque brachytherapy comprise a reduction in the number of goblet cells, moderate-to-severe stratification of the conjunctival epithelium, and stromal fibrosis [39]. These changes, which may also in part be due to conjunctival manipulation during plaque placement, are sufficient to explain the occurrence of dry eye symptoms in these patients. Treatment with topical lubricants or, if more severe, punctal plugs are indicated [29].

#### 3.3.2. Sclera

The sclera is a relatively radioresistant tissue [40]. Scleral complications include scleritis and, more rarely, scleral necrosis [19]. Inflammation manifests as deposits of migrating macrophages in the vicinity of the tumor at the level of the sclera and episclera and may occur in the context of associated autoimmune and infectious states [19,40]. Scleral necrosis has a reported incidence of 1–14.3% after brachytherapy, occurs after 5 to 351 months, and may range in severity from scleral thinning to globe perforation [41,42,43,44]. Risk factors include tumors located anterior to the equator, ciliary body invasion, extraocular extension, tumors thicker than 6 mm, radiation doses to the outer sclera of more than 400 Gy, increased intraocular pressure (IOP), inadequate conjunctival closure, disinsertion of the superior rectus muscle, and younger age [19,44,45]. An important differential diagnosis must be made with tumor recurrence. Slit-lamp examination, echography, and ultrasound biomicroscopy are useful in differentiating the two entities [41]. Berry et al. [45] reported three cases of conjunctival dehiscence and scleral necrosis occurring very soon, within 6 weeks from plaque therapy, near the site of muscle disinsertion. The authors propose, as possible mechanisms, a direct necrotizing effect of the irradiation, an atypical, milder form of surgically induced necrotizing scleritis (given the location at the site of muscle disinsertion), poor wound healing, inflammation related to tumor toxic syndrome, or an undiagnosed microinfection. All three patients responded to conservative therapy with topical antibiotics and steroids tapered over several weeks [45]. Other options in the management of scleral necrosis include observation, the use of lubricants, tissue glue, or if the necrotic area is extensive, surgical reconstruction with amniotic membrane, conjunctiva, scleral patch graft, dermal patch graft, or Tenon’s fascia [43,45].

#### 3.3.3. Iris

An irradiated iris can develop atrophy. Iris neovascularization is another important complication [19]. It usually results from the production of vascular endothelial growth factor (VEGF) by the ischemic retina or by the tumor itself. Iris neovascularization is often followed by neovascular glaucoma (NVG), an aggressive and usually refractory form of glaucoma, which frequently leads to vision loss and a painful eye and is an important reason for enucleation after radiotherapy [29].

#### 3.3.4. Intraocular Inflammation

Intraocular inflammation is common after irradiation and appears to be related to tumor necrosis, the direct irradiation of the ocular tissue, and the disruption of the blood-ocular barriers [3,46]. In a study by Lumbroso et al. [46], it was reported in 28% of patients up to 5 years after PBT. The inflammatory process usually consists of mild anterior uveitis with a favorable course under topical steroids and cycloplegics. Risk factors for its development are related to tumor characteristics, such as tumor height of more than 5 mm, tumor diameter of more than 12 mm, tumor volume of more than 0.4 cm^3^, and tumor location anterior to the equator [46].

#### 3.3.5. Lens and Cataract

The lens is the tissue with the highest radiosensitivity in the eye. Radiations with > 10 Gy result in the deformation of lens fibers, swallowing of replicating cells (with the formation of ‘Wedl cells’), and the subcapsular accumulation of debris, leading to cataract formation [25]. The reported incidence range and risk factors associated with cataracts are summarized in Table 1 and Table 2, respectively. The cataract is usually posterior subcapsular in nature, taking the form of vacuoles and scattered granules or, if radiation doses are high, it may present as a mature white cataract [25,29]. Cataract surgery is mainly aimed at allowing fundus visualization for tumor control and, to a lesser extent, at improving visual acuity (VA), as this remains limited by radiation optic neuropathy and retinopathy. Cataract surgery is usually successful using standard techniques and does not appear to increase the risk of metastasis [29,30].

#### 3.3.6. Vitreous Hemorrhage

Vitreous hemorrhage can be the result of neovessel rupture in the context of proliferative radiation retinopathy or the rupture of a retinal or a tumor vessel following direct tumor invasion of the retina and loss of the retinal barrier function [47]. Reported incidences are illustrated in Table 1. Vitreous hemorrhage is more common after brachytherapy, in tumors with a height of more than 5 mm, and in cases with posteriorly located melanomas [25,28,47]. Vitreous hemorrhage may clear spontaneously and observation usually represents the initial management [28,48]. If it is nonclearing or recurrent, vitrectomy may be indicated [25]. Vitrectomy was shown to be successful in clearing vitreous hemorrhage in 74% of cases after I-125 brachytherapy, and in 40% of cases, it resulted in an increase in VA of more than one line [48]. In a study by Tran et al. [47], vitrectomy was required in 2% of patients after PBT, having as indications the presence of vitreous hemorrhage, epiretinal membrane, tractional, rhegmatogenous or combined retinal detachment, and vitritis. The main roles of vitreoretinal surgery are facilitating tumor surveillance by allowing fundus visualization, increasing VA (for example, by clearing a vitreous hemorrhage or by repairing a macular hole), and preventing other complications (for example, preventing neovascular glaucoma by endoscopic panphotocoagulation). Vitrectomy does not appear to result in intraocular or distant tumor dissemination if tumor regression is achieved preoperatively [47,48,49].

#### 3.3.7. Retinal Complications—Retinal Detachment

Exudative retinal detachment often accompanies uveal melanoma and, in 90% of cases, clears within one year after irradiation of the tumor [49,50]. However, exudative retinal detachment can be a factor associated with the risk of local failure. Moreover, beyond 9 to 12 months, a nonclearing exudative retinal detachment accompanying an increase in tumor thickness may indicate a nonresponsive tumor and the need for additional treatment. These two parameters, the exudative retinal detachment and the tumor thickness, can thus be used in monitoring tumor response to treatment [50]. Post radiation therapy, retinal detachment may also develop into tractional, rhegmatogenous, or tractional–rhegmatogenous detachment. In these cases, retinal detachment is related to retinal thinning and atrophy, subretinal membranes, and atrophic retinal holes. Pars plana vitrectomy, which may be combined with scleral buckling or cataract removal, is the treatment of choice and leads to an improved VA in most patients [49].

#### 3.3.8. Retinal Complications—Radiation Retinopathy

Exposure to radiation results in multiple retinal changes. As photoreceptors do not replicate, they are intrinsically resistant to radiotherapy. However, the associated retinal changes, especially the intraretinal fluid, lead to associated photoreceptor atrophy [19]. The irradiated retinal pigment epithelium undergoes fibrous metaplasia, atrophy, and hyperplasia, clinically manifesting as alternating areas of hyper- and hypopigmentation [19]. Irradiated retinal vessels display an altered endothelium with subsequent capillary occlusion and associated areas of increased capillary permeability. This results in retinal ischemia, formation of collateral vessels, telangiectasias, and microaneurysms, leading to the characteristic changes of radiation-induced retinopathy, namely, retinal neovascularization, macular edema, exudative retinal detachment, hard exudates, cotton wool spots, vitreous and retinal hemorrhages, and retinal degeneration [8,19,29].

Using ultra-wide-field fluorescein angiography, McCannel et al. [51] proposed a classification of radiation retinopathy in patients after I-125 brachytherapy:

- Grade zero: no vascular abnormality except in the tumor area (no retinal vascular leakage);
- Grade one: late foveal leakage;
- Grade two: grade one plus peripheral vascular leakage;
- Grade three: grade two plus nonperfusion greater than one disc area in the midphase;
- Grade four: grade three plus retinal neovascularization.

The severity correlates with younger age, time from treatment, progression of macular changes on optical coherence tomography (OCT), development of NVG, and decrease in VA, which supports the fact that radiation retinopathy is a progressive ischemic disease [51]. The reported incidence range and risk factors associated with radiation retinopathy are summarized in Table 1 and Table 2, respectively.

#### 3.3.9. Retinal Complications—Radiation Maculopathy

Radiation maculopathy is related to vascular endothelial damage and resembles diabetic retinopathy, manifesting as capillary nonperfusion, the presence of telangiectasias and microaneurysms, hemorrhages, exudates, cotton wool spots, atrophy or neovascularization, and macular edema [52]. The reported incidence range and risk factors associated with radiation maculopathy are summarized in Table 1 and Table 2, respectively.

Horgan et al. [52] conceived a classification of macular edema based on OCT findings that correlate with VA:

1. Noncystoid extrafoveal;
2. Cystoid extrafoveal;
3. Noncystoid foveal;
4. Mild or moderate cystoid foveal;
5. Severe cystoid foveal.

Some authors added a sixth grade in the classification, representing severe cystoid foveal edema with associated subretinal fluid [53].

Prophylactic measures include photocoagulation of the ischemic peripheral retina, intravitreal administration of anti-VEGF agents (bevacizumab and ranibizumab), and subtenon triamcinolone acetonide [53]. The best choice for treating macular edema is at present anti-VEGF agents and corticosteroids (triamcinolone acetonide and dexamethasone implant) administered intravitreally, which have replaced laser photocoagulation. Regression of macular edema is usually accompanied by an increase in VA. All anti-VEGF agents (bevacizumab, aflibercept, ranibizumab) have shown their efficiency in obtaining good anatomical results and preventing vision loss. Long-term treatment and an interval between doses of less than 3 months have shown better outcomes [53]. An alternative to anti-VEGFs are intravitreal corticosteroids. Apart from its anti-inflammatory effects, triamcinolone appears to improve the function of Muller cells, thus stimulating fluid clearance from the macular region [54]. The dexamethasone implant has shown sustained and significant reduction in the central subfield thickness and central foveal thickness in the first three months alongside an improvement in VA. However, its effect diminishes after 4 months, and retreatment is required [55]. Complications related to corticosteroid administration include increased IOP and cataract formation [53].

#### 3.3.10. Choroid

Choroidal complications comprise the same vascular changes found in the retinal vasculature, with occlusion, formation of microaneurysms, telangiectasias, and neovascular membranes [3,19].

#### 3.3.11. Optic Neuropathy

Radiation doses of more than 50 Gy lead to optic neuropathy by direct and ischemic mechanisms. Irradiation results in damage to glial cells, leading in time to demyelination and neural degeneration [29]. Endothelial cell damage, as in the case of retinopathy, leads to vascular occlusion and consecutive ischemia. Radiation optic neuropathy is characterized by profound visual loss usually occurring 1.5–2 years after radiation therapy [19,25]. The reported incidence range and risk factors associated with radiation optic neuropathy are summarized in Table 1 and Table 2, respectively. The optic nerve is most susceptible to damage in the 2 mm of its length that are most proximal to the retina. This is considered to be the result of the lack of myelin at this level and because this part of the optic nerve is found at the border between the territories of the retinal and choroidal networks. If the optic nerve is included in the irradiation field and receives high or even full doses of radiation, modulating the radiation dose along the length of the optic nerve can help retain some visual function [34]. There are reported cases of some spontaneous improvement in VA. Shields et al. [56] attempted the treatment of radiation papillopathy, manifesting as an elevated, hyperemic disc with surrounding hemorrhages with an intravitreal injection of triamcinolone acetonide (4 mg/0.1 mL). There was an initial improvement in seven patients and a stable or better visual acuity at 11 months with the resolution of clinical signs. Hyperbaric oxygen therapy has also been tried in order to break the ischemia–necrosis cycle. However, apart from a few sporadic cases, hyperbaric oxygen therapy has not been proven to improve visual outcomes [57]. In most cases, radiation-induced optic neuropathy progresses to optic atrophy and irreversible vision loss.

#### 3.3.12. Secondary Glaucoma—Neovascular Glaucoma

Glaucoma can be tumor-related or radiation-induced. Irradiation may lead to glaucoma by angle fibrosis or angle neovascularization. In a study by Puusaari et al. [21], of all the secondary glaucomas, 84% were neovascular (NVG), 10% were by secondary angle closure, and 6% had an open angle mechanism [21]. The reported incidence range and risk factors associated with secondary and, in particular, with neovascular glaucoma are summarized in Table 1 and Table 2, respectively. The first line of therapy is topical medication. There is, however, a discussion regarding the use of prostaglandin analogs which increase the uveoscleral outflow and may thus theoretically favor metastases. Cyclodestructive procedures are the next option, as they decrease aqueous production. Filtrating procedures are less desirable for two reasons. Firstly, they are only indicated in eyes where tumor clearance was successful; otherwise, they may increase the risk of metastasis [58]. Secondly, irradiation leads to tissue fibrosis, which makes surgery more difficult and more predisposed to failure [58]. Nevertheless, there are studies that report successful IOP management with trabeculectomy (success rate 78.6%) and Baerveldt shunt implantation (success rate 86%), the latter not being associated with any sight-threatening complications, enucleations due to ocular hypertension, local tumor recurrences, or metastases related to the procedure [58,59].

NVG is the most important cause of secondary enucleation [58,60]. Mechanisms involved in the development of NVG are radiation retinopathy, characterized by retinal ischemia, and toxic tumor syndrome, both of which result in the production of proangiogenic factors [61]. Iris neovascularization may also be promoted by anterior segment ischemia resulting from microvascular iris ischemia, disinsertion of horizontal rectus muscles in brachytherapy, or irradiation of the long posterior ciliary vessels [62]. In a study by Mahdjoubi et al. [63], bevacizumab administered as a series of three monthly intravitreal injections has proven useful as a preventive measure and in the initial phases of NVG before neovascularization reached the anterior chamber angle. However, it was inferior to panretinal photocoagulation in reducing the rate of enucleation [63]. Panretinal photocoagulation was shown to reduce the enucleation rate by eliminating the angiogenic stimulus represented by the ischemic retina. In cases of extensive retinal detachment, panretinal photocoagulation should be deferred until the resorption of the subretinal fluid. In the meantime, anti-VEGF agents can be used to delay the emergence of anterior segment neovascularization [11,63]. Medical therapy is usually inefficient in controlling the IOP in NVG. Surgical options include cyclophotocoagulation, glaucoma drainage devices, and trabeculectomy. Two other interesting methods have also been explored, the drainage of subretinal fluid with the aim of reducing retinal ischemia and tumor resection that prevents toxic tumor syndrome. Both procedures were shown to reduce the rate of enucleation [60].

#### 3.3.13. Toxic Tumor Syndrome

Toxic tumor syndrome is the result of residual irradiated tumor tissue in which proinflammatory and angiogenic factors are being synthetized. This causes persistent intraocular inflammation and additional stimuli for neovascularization of the anterior segment. This, in addition to the neovascular trigger represented by the ischemic irradiated retina, may result in NVG [25]. Toxic tumor syndrome is more pronounced with large, bulky tumors and in the presence of extensive retinal detachment [15]. Treatment options include intravitreal administration of anti-VEGF agents or steroids. Toxic tumor syndrome can be prevented by reducing the tumor volume via resection or transpupillary thermotherapy [25].

#### 3.3.14. Tumor-Related Lipid Exudation

Tumor-related lipid exudation is a condition appearing in the setting of posterior uveal melanoma that is associated with poor visual prognosis, increased risk of other ocular complications, and a higher rate of enucleation [64]. The pathogenesis seems to be mainly related to residual tumor tissue alongside radiation-related incompetence of blood vessels in the tumor or the surrounding tissues and abnormal serum lipids [64,65]. The incidence of tumor-related lipid exudation appears to be influenced mainly by tumor characteristics while radiation parameters seem to have little effect [64]. Tumor-related lipid exudation is associated with younger age, large tumor size, tumor rupture of Bruch’s membrane, and anomalies of serum lipids, more specifically high levels of low-density lipoproteins and low levels of high-density lipoproteins [65,66]. Tumor-related lipid exudation also occurs more frequently in association with other ocular complications, such as iris neovascularization, NVG, posterior synechia, vitreous and subretinal hemorrhage, retinal detachment, and retinoschisis over the tumor. Rupture of Bruch’s membrane, which is highly prevalent in patients who develop tumor-related lipid exudation, may allow blood and its components, including VEGF, to reach the retina and vitreous, which may explain the associations with the other complications. Irradiation-induced tumor vasculopathy manifesting with intratumoral hemorrhages and extensive subretinal lipid exudation appearing relatively soon after radiotherapy (6–9 months) is associated with chronic retinal detachment and a poor visual prognosis [50]. Prevention and treatment should be aimed at reducing the residual tumor tissue (surgical resection or transpupillary thermotherapy) or at neutralizing the released mediators (intravitreal anti-VEGF agents) [64].

#### 3.3.15. Sympathetic Ophthalmia

Sympathetic ophthalmia is a very rare but potentially blinding complication of radiation therapy for UM. A risk of 6.1 in 1000 of developing sympathetic ophthalmia after PBT has been reported [61]. However, visual recovery is possible with prompt diagnosis and appropriate treatment. Treatment regimens usually consist of high-dose intravenous corticosteroids for three days followed by oral administration beginning at 1 mg/kg/day and slow tapering. Other immunosuppressants may be added to the regimen. Topical corticotherapy is also associated [61].

#### 3.3.16. Ocular Adnexa

The eyelids can be affected if PBT is not performed with the eyes closed. Potential complications include skin scars and depigmentation, madarosis and scarring, and metaplasia of the conjunctiva and the eyelid margins [3].

Lacrimal system complications appear when its different components are part of the irradiation field in PBT. For nasally located tumors, irradiation may lead to punctal and canalicular inflammation and scarring, leading to persistent epiphora. Prophylactic measures include topical steroids and lacrimal stenting. Treatment is usually surgical [67]. For temporally located tumors, irradiation of the lacrimal gland with subsequent atrophy may result in keratoconjunctivitis sicca [3]. Artificial tears are required in such cases.

Although they are relatively protected from irradiation by the treatment delivery design, the extraocular muscles present ultrastructural changes after exposure to radiation [29]. After plaque brachytherapy, some patients complain of transient diplopia, resolving within months after the treatment, and it is unclear whether this is the result of irradiation or of muscle manipulation during plaque insertion. If persistent ocular alignment or motility occurs, the use of prisms, injection of botulinic toxin, or even strabismus surgery may be indicated [29,68].

High-dose (60 Gy) radiation therapy can be applied to the eye socket as adjuvant therapy after enucleation in high-risk patients, such as those with vortex vein invasion or trans-scleral extension. In these cases, it can lead to severe socket contraction that prevents prosthesis wear in about 40% of patients. This complication can be managed surgically, by releasing scar tissue and reconstructing the eye socket with good postoperative results [69].

#### 3.3.17. Decrease in Visual Acuity

Preserving a useful VA is one of the most important aims of the treatment of UM. However, in most cases, the visual outcome is poor. A final VA of less than 20/200 was reported in 23–87% of cases after plaque therapy [8], in 33–86% of cases after PBT [8], and in 60–65% of cases after SRS [4,7]. Both radiation retinopathy and optic neuropathy are associated with a poor visual outcome, and the dose of radiation applied to the macula and the optic nerve is related to the risk of low vision and blindness [32,36]. The COMS identified the following as risk factors for vision loss: increased tumor thickness, a tumor that is not dome-shaped, tumor location near to or at the level of the macula, and secondary retinal detachment [12]. Other factors related to decreased vision are hypertension, age, reduced VA at diagnosis, tumor height, and largest basal diameter, tumor distance from the fovea and the optic nerve, ciliary body involvement, retinal invasion, extraocular invasion, posterior tumor location, radiation dose, and length of the optic nerve irradiated [8,20,34,70].

#### 3.3.18. Enucleation due to Complications

After radiation therapy for UM, enucleation may be necessary in the case of resistance to treatment, local recurrence, and complications, such as phthisis bulbi and a blind and painful eye [58]. Reported enucleation rates are variable, between 3 and 15% after Ru-106 plaque therapy [27,28], between 1 and 6.8% after I-125 brachytherapy [71,72], between 4 and 26% after PBT [5,8], and between 7 and 23% after SRS [31,73]. Among complications, NVG is the leading cause of enucleation [58,60]. Around 50% of the eyes that develop NVG require enucleation [11,71]. Factors associated with higher rates of enucleation after SRS are large tumors (largest basal diameter greater than 18 mm, T4 in the TNM classification), tumors involving the ciliary body, the presence of persistent exudative retinal detachment, and NVG [24].

### 3.4. Reducing Complication Rates

Reducing complication rates can be achieved by lowering the dose of radiation, adjusting safety margins by the use of eccentric and customized plaques and treatment planning in order to protect structures vital to maintaining visual function, and using neoadjuvant and adjuvant methods in order to reduce tumor volume [38]. Ru-106 and I-125 are the most used isotopes in plaque therapy today, as they have lower energy and are thus associated with a lower risk of complications [74].

Regarding radiation doses, in I-125 plaque therapy, a dose of less than 85 Gy to the tumor apex appears to achieve good local tumor control with better preservation of the visual function and lower enucleation rates. For tumors close to the macula, it may be necessary to use doses of less than 70 Gy to the tumor apex, especially if the contralateral eye has poor vision [75]. In Gamma Knife radiosurgery, a median marginal dose of 22 Gy achieves good local tumor control with lower rates of complications including radiation retinopathy and NVG [6]. Other authors using Gamma Knife advocate for a limitation of the radiation applied to the optic nerve at 13.2 Gy in order to reduce the risk of blindness while a dose of less than 8.5 Gy was found to delay the onset of VA deterioration [35]. A study conducted by Murray and associates showed that for tumors of less than 5 mm in height treated with brachytherapy, applying treatment to the actual tumor height instead of the 5 mm standard used by the COMS resulted in similar local tumor control, rate of metastases, and visual outcomes but was associated with a significantly lower overall complication rate, especially that of radiation retinopathy and cataract formation [74]. In PBT, a wedge filter can be used to create a high, more uniform radiation dose to the tumor tissue and a lower dose to structures vital to vision [34].

Since the impact of ocular treatment on survival is uncertain, in selected cases, such as monophthalmic patients, the safety margins can be adjusted [76]. This is usually considered an option in tumors that are close to the macula or the optic nerve where the inclusion of these structures into the radiation field is likely to lead to vision loss. In plaque radiotherapy, this can be achieved by the use of eccentric plaques whose posterior margin is aligned with the posterior margin of the tumor. If correctly positioned, eccentric plaques achieve good local tumor control [77]. Similarly, the recommended 2.5 mm safety margins for PBT can be decreased provided that the tumor is not diffuse, the appropriate positioning during treatment can be achieved and maintained, and the patient accepts the theoretically increased risk of local tumor recurrence and gives informed consent [15].

In the case of large tumors, radiotherapy can be associated with tumor resection or transpupillary thermotherapy in order to reduce tumor volume and lower the risk of complications related to radiation. This results, on the one hand, from reducing the need for high doses and, on the other hand, from eliminating tumor tissue that produces cytokines promoting inflammation and neovascularization [18,78]. Preirradiation surgical resection is, however, controversial and reserved for patients who are highly motivated to preserve vision but whose tumors are very close to the disc margin, so any form of radiation therapy would cause optic neuropathy [15].

## 4. Conclusions

Eye-sparing radiotherapy is the first line of treatment for small, medium, and selected large uveal melanomas. The pathophysiology of radiation-induced complications resides firstly in the direct cytotoxic effect of radiation on the tissues surrounding the tumor and on the tumor itself, manifesting as toxic tumor syndrome. Secondly, irradiation damages the endothelium of blood vessels, resulting in ischemic changes. The most frequent radiation-induced complications are cataracts, which may be managed surgically, and radiation retinopathy. Radiation retinopathy and radiation optic neuropathy are the main complications associated with a poor visual outcome, which is common in most treated eyes. Around half of secondary enucleations are due to complications, especially neovascular glaucoma. Efforts for reducing the occurrence and impact of radiation-related complications focus on reducing radiation doses while maintaining the same tumor control rate. This may be achieved by customized treatment planning, taking into account the dimensions, location, and proximity of the tumor to structures vital to maintaining vision, and by associating radiotherapy with other methods, such as tumor resection and transpupillary thermotherapy, in order to reduce the need for high radiation doses and eliminate the remaining tumor tissue, which is a source of proinflammatory and angiogenetic factors.

## Figures and Tables

**Table 1:** Reported complication rates after different radiation therapies.

Complication	Ru-106 Brachytherapy	I-125 Brachytherapy	PBT	SRS
Cataract	4.2–53.8%	8–69%	20–62%	15–67.8%
Rubeosis iridis	4.8–12%	4–19%	12–45%	-
Secondary glaucoma	2–12%	-	7–30%	5.6–15.2%
Neovascular glaucoma	10%	2–45%	11.7–23%	3–35%
Vitreous hemorrhage	12.7–15%	3.1–36%	9–14%	4–14.4%
Retinal detachment	17.4%	7.3–25%	38%	-
Radiation retinopathy	20–53%	10–62.8%	23–68.1%	5–44%
Radiation maculopathy	19.6–50%	13–52%	30–66.5%	9–30%
Optic neuropathy	2–32.8%	3.6–46%	7–47.5%	9–41%

I = iodine; PBT = proton beam therapy; Ru = ruthenium; SRS = stereotactic radiosurgery.

**Table 2:** Risk factors associated with the development and time to the development of different complications.

Complication	Ru-106 Brachytherapy	I-125 Brachytherapy	PBT	SRS
Cataract	-tumor height > 5 mm -increased LBD -anterior location -radiation dose to tumor apex > 90 Gy [27,28]	-increased tumor height -LBD > 10 mm -high radiation dose to the lens -male gender -age > 65 years [21,29]	-tumor close to the optic nerve -high radiation dose to the lens -advanced age [30]	-tumor dimensions (T3 or T4 in the TNM classification) -tumor further from the fovea/anteriorly located -high radiation dose to the lens [4,31]
Rubeosis iridis	-LBD > 15 mm [28]	-increased tumor height -disinsertion of horizontal rectus muscle -high radiation dose to the opposite retina [29,32]	-	-
Secondary glaucoma	-mushroom-like shape -LBD > 15 mm -distance between tumor and disc margin > 10 mm [27,28]	-increased tumor thickness -IOP at diagnosis -pretreatment exudative retinal detachment -increased radiation dose to the opposite retina [21,32,33]	-	-increased tumor thickness [33]
Neovascular glaucoma	-LBD > 15 mm -TNM class T3 and T4 [28]	-increased tumor height [29]	-proximity to the papilla [34]	-increased tumor thickness >7.4 mm [6]/> 8.7 mm [35] -less pigmented UM -Bruch’s membrane rupture -the volume of the posterior pole receiving > 20 Gy -peripapillary location -anteriorly located tumor [4]
Radiation retinopathy	-increased radiation dose to tumor apex [27]	-increased tumor thickness -tumor location -higher radiation dose -younger age [26,29]	-tumor less than 2.5 mm from macula -increased tumor thickness [36]	-tumor located in the macular region -reduced distance between tumor and optic disc -radiation dose > 14.9 Gy -diabetes mellitus -younger age [6,35,37]
Radiation maculopathy	-mushroom-like shape -increased tumor height and volume -distance between tumor margin and fovea < 2 mm -radiation dose to fovea >50 Gy -subretinal fluid -diabetes mellitus [27,28,38]	-tumor height > 4 mm -increased LBD -radiation dose to macula > 90 Gy -proximity of tumor to foveola -male gender -younger age [26,29]	-tumor proximity to fovea -high radiation dose to fovea [36]	-
Optic neuropathy	-distance between tumor and disc < 1.5 mm/1 DD -increased LBD [27,28]	-dose to optic nerve > 55 Gy -distance between tumor and optic disc < 4 mm -increased LBD -ciliary body involvement [21,29]	-tumor proximity to papilla -high radiation dose to the optic disc [36]	-tumor close to the papilla -high radiation dose to the optic nerve -distance of the optic nerve from the prescription isodose [6,35]

DD = disc diameter; I = iodine; Gy = Gray; IOP = intraocular pressure; LBD = largest basal diameter (of the tumor); PBT = proton beam therapy; Ru = ruthenium; SRS = stereotactic radiosurgery.

## Data Availability

Not applicable.
